# Case report of pregnancy complicated by severe pulmonary hypertension from mitral stenosis and placenta accreta spectrum disorder: management of two life-threatening conditions

**DOI:** 10.1093/ehjcr/ytae055

**Published:** 2024-01-29

**Authors:** Jaya Batra, Mirella Mourad, Fady Khoury Collado, Koji Takeda, Andrew Greenwald, Laurence Ring, Xuxin Chen, Jennifer Haythe

**Affiliations:** Division of Cardiology, Department of Medicine, Columbia University Vagelos College of Physicians and Surgeons, 630 W 168th St, PH 12 STEM, New York, NY 10032, USA; Department of Obstetrics and Gynecology, Columbia University Vagelos College of Physicians and Surgeons, 630 W 168th St, New York, NY 10032, USA; Department of Obstetrics and Gynecology, Columbia University Vagelos College of Physicians and Surgeons, 630 W 168th St, New York, NY 10032, USA; Division of Cardiac, Thoracic, and Vascular Surgery, Department of Surgery, Columbia University Vagelos College of Physicians and Surgeons, 630 W 168th St, New York, NY 10032, USA; Department of Anesthesiology, Columbia University Vagelos College of Physicians and Surgeons, 630 W 168th St, New York, NY 10032, USA; Department of Anesthesiology, Columbia University Vagelos College of Physicians and Surgeons, 630 W 168th St, New York, NY 10032, USA; Department of Pediatrics, Columbia University Vagelos College of Physicians and Surgeons, 630 W 168th St, New York, NY 10032, USA; 1 Division of Cardiology, Department of Medicine, Columbia University Vagelos College of Physicians and Surgeons, 630 W 168th St, PH 12 STEM, New York, NY 10032, USA

**Keywords:** Valvular heart disease, Cardio-obstetrics, Pregnancy, Extracorporeal membrane oxygenation, Case report

## Abstract

**Background:**

Antenatal cardiovascular disease is a major cause of maternal morbidity and mortality. Severe rheumatic mitral stenosis is especially poorly tolerated during pregnancy.

**Case Summary:**

We present a young woman with severe pulmonary hypertension secondary to rheumatic mitral stenosis. She presented at 25 weeks 4 days gestation for evaluation of a pregnancy complicated by placenta accreta spectrum disorder. Invasive hemodynamic testing was carried out to delineate her hemodynamics, and a multidisciplinary cardio-obstetrics team collaborated closely with the patient and her partner to create a management plan. Ultimately, the patient was initiated on veno-arterial extracorporeal membrane oxygenation and underwent caesarean section delivery followed by hysterectomy and subsequent valve replacement surgery.

**Discussion:**

This case describes the treatment options considered to balance the risk of decompensation in the setting of severe pulmonary hypertension with hemorrhage associated with placenta accreta spectrum disorder. It highlights the importance of a multidisciplinary, team-based approach to the management of high-risk cardiac conditions throughout pregnancy.

**Learning points**

- To understand the hemodynamic consequences of mitral stenosis during pregnancy.
- To understand the indications for intervention and treatment options for significant mitral stenosis in the setting of pregnancy.

## Primary specialties involved in addition to cardiology

- Department of Obstetrics and Gynecology
- Department of Cardiac Surgery
- Department of Anesthesiology
- Department of Pediatrics

## Introduction

Globally, antenatal cardiovascular disease is a leading cause of maternal and neonatal complications during pregnancy. In countries where rheumatic fever is endemic, rheumatic heart disease accounts for >70% of maternal heart disease.^1,2^ Rheumatic mitral stenosis (MS) is especially poorly tolerated due to the hemodynamic changes that occur during pregnancy and is associated with high maternal and fetal morbidity. This case illustrates the hemodynamic consequences of MS during a pregnancy complicated by placenta accreta spectrum disorder (PASD) and highlights important considerations for the treatment of significant valvular heart disease in the setting of an already complicated pregnancy.

## Summary figure

**Table:** 

Days from admission	Patient’s clinical course
**1**	Thirty-one-year-old woman at 26 weeks gestation with severe pulmonary hypertension secondary to rheumatic mitral stenosis presented for management of a pregnancy complicated by placenta accreta spectrum disorder.
**2**	Pulmonary artery catheterization demonstrated severely elevated filling pressures. She did not tolerate medical therapy. The cardio-obstetrics team met with the patient and her partner to determine next steps.
**3**	Brought to the operating room (OR) for veno-arterial extracorporeal membrane oxygenation (VA-ECMO) supported caesarean delivery and hysterectomy. Extubated in the OR. Her infant was taken to the Neonatal Intensive Care Unit for further management.
**6**	Returned to the OR for surgical aortic and mitral valve replacement and tricuspid valve annuloplasty. Decannulated from ECMO in the OR.
**7**	Extubated
**19**	Discharged home from the hospital
**Follow-up**	At 30 days from the date of admission, the patient was doing well at home with her family. Her infant, who was born at 25 weeks 6 days, was discharged from the hospital at postmenstrual age 38 weeks 5 days.

## Case presentation

A 31-year-old gravida three para 1011 (i.e. one term delivery, one abortion, and one living child) woman at 25 weeks and 4 days of gestation was referred to our hospital for evaluation and management of a pregnancy complicated by severe MS and PASD. As a child in Bangladesh, she had acute rheumatic fever complicated by rheumatic heart disease. Her obstetric history was notable for two previous pregnancies, including a termination by dilation and curettage procedure in Bangladesh (year unknown) and a caesarean delivery under general anaesthesia with her first child in 2017. She reported having undergone two previous percutaneous mitral balloon valvuloplasties (PMBV) for MS, the first at age 12 and the second in 2019 at age 28 for severe MS with a mean mitral valve gradient of 18 mmHg which was described as ‘unsuccessful.’ At that time, she was advised to pursue valve replacement surgery.

She did not follow-up with medical care and presented to an academic tertiary care institution four years later early in her third pregnancy. There, she had magnetic resonance imaging concerning for PASD with invasion into the bladder and parametria. In addition, a transthoracic echo (TTE) showed severe MS with peak and mean mitral valve gradients of 46 and 29 mm Hg, respectively, and a severely elevated estimated right ventricular systolic pressure (RVSP) of 90 mmHg.

Because of the obstetric and cardiac complexities of the case, the patient was transferred to the cardiac intensive care unit of our hospital, a quaternary care centre in New York City. She reported no symptoms before becoming pregnant but over the last several weeks had experienced episodes of lightheadedness and palpitations. Medications before hospitalization included metoprolol succinate 25 mg daily and furosemide 20 mg daily. On physical exam, the patient had blood pressures ranging from 80–112 systolic/58–79 diastolic mmHg, a heart rate of 90–117 b.p.m. in sinus rhythm, a respiratory rate of 18–34 breaths per minute, and a peripheral oxygen saturation of 93–100% on ambient air. She had jugular venous distension to the jawline, a 2/4 rumbling diastolic murmur auscultated over the area of apical impulse, a 3/6 systolic murmur auscultated throughout the precordium, a prominent P2 without parasternal heave, bibasilar crackles, and warm extremities without peripheral edema. On bedside obstetric sonogram, an active fetus was noted, and PASD was confirmed.

**Figure 1:**
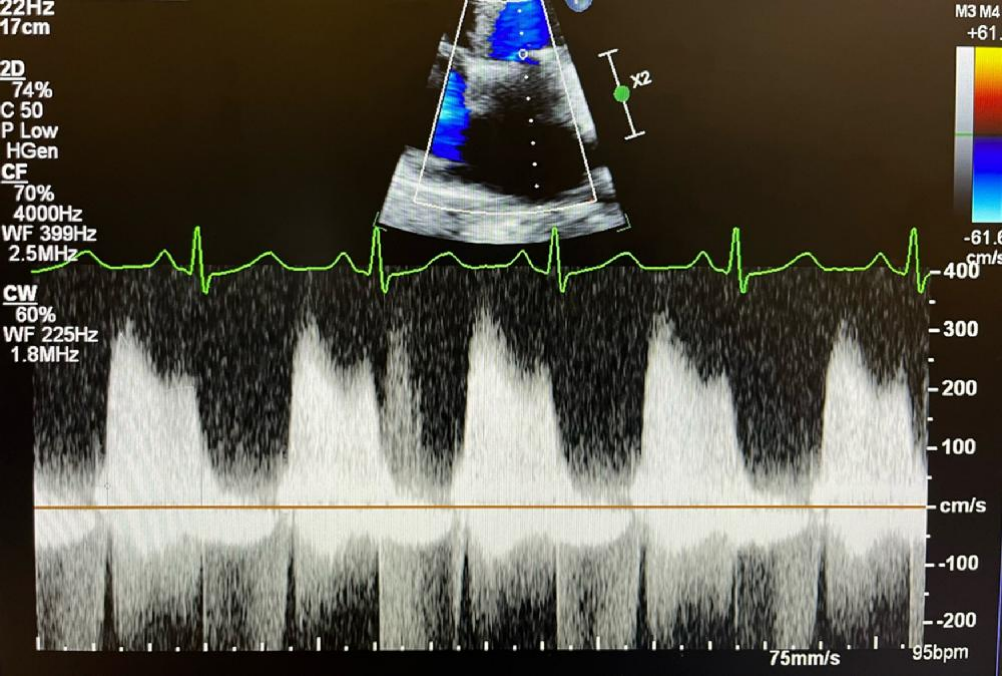
Pre-operative transthoracic echocardiography imaging, continuous wave Doppler parallel to the mitral inflow in apical four-chamber view. Peak and mean mitral valve gradients are measured as 34 and 19 mmHg at a heart rate of 95 b.p.m., respectively.

Laboratory testing on admission was notable for a hemoglobin of 9.4 g/dL and an N-terminal prohormone of brain natriuretic peptide of 613 pg/mL (normal range <450 for age <50 years). A TTE showed heavily calcified mitral and aortic valves with severe MS (peak and mean mitral valve gradients of 34 and 19 mmHg at a heart rate of 95 b.p.m., respectively, mitral valve area 0.9 cm2 by planimetry, Wilkins score 12), moderate aortic stenosis (AS) (peak velocity 3.6 m/s, mean gradient 31 mmHg), mild mitral and aortic regurgitation, and severe tricuspid regurgitation (Supplementary material online, *Videos 1 and 2*, *Figure 1*). The left ventricular end-diastolic diameter was 4.4 cm, end-systolic diameter was 2.9 cm, left ventricular mass indexed to body surface area was 85 g/m2, and the ejection fraction was 60–65%. The right ventricle was severely dilated with end-diastolic diameters of 5.1 and 3.7 cm at the base and mid-ventricle, respectively. The right ventricular peak systolic excursion velocity was 11 cm/s and the tricuspid annular plane systolic excursion was 1.8 cm. The left atrial volume indexed to body surface area was 56 mm/m2. The RVSP was estimated as 87 mmHg.

A pulmonary artery catheter placed to characterize the patient’s hemodynamics demonstrated severe pulmonary hypertension with a right atrial pressure of 19 mmHg, systolic and diastolic pulmonary artery pressures of 82 and 43 mmHg, respectively, a pulmonary capillary wedge pressure of 37 mmHg, and a cardiac index of 2.9 L/m2. The transpulmonary gradient was 19 mmHg and the pulmonary vascular resistance was 4 Wood units, consistent with both pre- and post-capillary pulmonary hypertension. Intravenous furosemide 20 mg and metoprolol tartrate 6.25 mg every 6 hours was initiated. However, she quickly developed symptomatic hypotension in response to these therapies, so they were discontinued.

A multidisciplinary cardio-obstetrics team which included physicians from the divisions of advanced heart failure, cardio-thoracic surgery, maternal-fetal medicine, gynecologic oncology, cardiac and obstetric anaesthesiology, neonatal intensive care, and consult liaison psychiatry met with the patient and her partner to determine next steps. Because of the presence of PASD, the risk of significant blood loss during delivery and potential requirement for large volume transfusions was estimated as extremely high. Accounting for the patient’s potential for significant blood loss and her rheumatic heart disease with severe MS, secondary pulmonary hypertension, and right ventricular dysfunction, urgent caesarean delivery with probable hysterectomy followed days later by surgical replacement of the mitral and aortic valves was recommended to the patient. Because significant hemodynamic fluctuations were anticipated during surgery, a plan was made for the initiation of veno-arterial extracorporeal membrane oxygenation (VA-ECMO) for right ventricular support should signs of hemodynamic instability develop. She was counselled extensively on the risks of maternal morbidity and mortality, the risks of fetal complications with delivery at an extreme preterm gestational age, and the risks of cannula and bleeding complications. She expressed an understanding of these risks. In addition, while mechanical aortic and mitral prosthetic valves were strongly recommended to the patient, she repeatedly expressed that she did not want to manage long-term warfarin therapy and therefore had a strong preference for bioprosthetic valve replacements.

On day three of the hospitalization, she was brought to the operating room (OR). A combined spinal-epidural anaesthetic was placed after which she developed significant hypotension necessitating initiation of norepinephrine at 10 mcg/min and vasopressin at 4 units/h. A decision was made to proceed with cannulation to peripheral femoro-femoral VA-ECMO. Neuraxial anaesthesia was inadequate, so she was converted to general anaesthesia. A transesophageal echocardiogram was performed which showed severe MS with a mean gradient of 11 mmHg (on VA-ECMO), moderate to severe AS, left ventricular ejection fraction of 30%, and a moderately dilated right ventricle with reduced function (Supplementary material online, *Video 3*). She then underwent successful VA-ECMO supported caesarean delivery of a vigorous male infant at 25 weeks 6 days gestation followed by an uncomplicated abdominal hysterectomy and bilateral salpingectomy with an estimated blood loss of 500 mL. She was extubated and brought to the cardio-thoracic intensive care unit for 48 hours.

On post-operative day 3, she underwent surgical bioprosthetic aortic and mitral valve replacement and tricuspid valve annuloplasty without complication. She was decannulated from VA-ECMO in the OR and extubated the next day. The infant, who was born at 25 weeks 6 days, was discharged from the hospital at postmenstrual age 38 weeks and 5 days. He had no supplemental oxygen requirement and was exclusively feeding by mouth with cranial ultrasounds reassuring against intraventricular hemorrhage. The patient’s TTE prior to discharge showed a bioprosthetic aortic valve which was well-seated without stenosis (peak velocity 2 m/s) or regurgitation, a bioprosthetic mitral valve that was functioning normally with a mean transvalvular gradient of 4 mmHg at a heart rate of 80 b.p.m., and a repaired tricuspid valve with moderate regurgitation. Left ventricular size and function were normal, the right ventricle was moderately increased in size with normal function, and the pulmonary artery systolic pressure was estimated to be 36 mmHg. She was initiated on daily low-dose aspirin and an oral anticoagulant for episodes of post-operative atrial fibrillation and surgical bioprosthetic valve implantation with plans to re-evaluate the need for anticoagulation after three months. The patient wished to breastfeed. Because of limited data on the safety of direct oral anticoagulants while breastfeeding, warfarin was preferred with a goal international normalized ratio of 2–3. She was also started on a heart failure regimen with metoprolol succinate 25 mg, spironolactone 25 mg, and furosemide 20 mg.

## Discussion

This case highlights several important points that relate to the current European Society of Cardiology (ESC) guidelines, including: (1) the importance of a multidisciplinary, team-based approach to the management of high-risk cardiac conditions throughout pregnancy, and (2) the indications for intervention and treatment options for significant MS in the setting of pregnancy.^3^

Placenta accreta spectrum disorder refers to abnormal attachment of the placenta to the uterus and is one of the most dangerous conditions in pregnancy due to the risk of life-threatening bleeding.^4^ Transfusion at the time of delivery is reported in up to 50% of patients.^5,6^ The presence of PASD in addition to severe pulmonary hypertension in this case greatly impacted clinical decision-making, particularly the need for hysterectomy before valvular surgery.

Significant hemodynamic changes occur during pregnancy which allow the maternal cardiovascular system to maintain adequate uteroplacental circulation.^7^ These include a fall in systemic vascular resistance due to both systemic vasodilation and the development of a low resistance uteroplacental circuit, an increase in resting heart rate, and expansion of total maternal blood volume. As was the case for the patient described here, these cardiovascular changes can unmask previously asymptomatic disease. In the presence of severe MS, expansion of total blood volume and reduced diastolic filling time due to an increase in heart rate further increase already elevated left atrial and pulmonary pressures.^8^ Heart failure symptoms have been reported to develop in up to 50% of women with severe MS over the course of pregnancy, most commonly during the second trimester.^9^

For women with known MS, pre-pregnancy planning is critical. Current ESC guidelines recommend that all women with significant MS be counseled against pregnancy until intervention is considered, even if asymptomatic, especially if the mitral valve area is <1 cm^2^ [class of recommendation (COR): I, level of evidence (LOE): C].^3^ For women requiring intervention, PMBV is the first-line treatment modality. Surgical valve repair or replacement may be considered when PMBV is not feasible.

While intervention before pregnancy is highly recommended, it is not uncommon particularly in low and middle-income countries for women to be unaware of the presence of significant MS until they seek medical attention for new symptoms of decompensated heart failure during pregnancy. As was highlighted in the case presented, clinical decision-making in these settings is particularly challenging. Medical therapy including beta-1-selective blockers and diuretics should be trialed (COR: I, LOE: B).^3^ For women with persistent New York Heart Association III-IV symptoms and/or pulmonary artery systolic pressure ≥50 mmHg despite medical treatment, intervention may be considered with percutaneous strategies preferred due to the high risk of fetal complications reported with open-heart surgery (COR: IIa, LOE: C).^3,10^ Management in these cases will be highly dependent on centre-specific availability of resources and expertise, patient factors, and the priorities of the mother.

In this case, three management options were considered. The first option was expectant management followed by caesarean delivery at term. However, the patient already had signs of decompensated heart failure which were expected to worsen as total maternal blood volume and resting heart rate continued to rise during the third trimester. Additionally, she had demonstrated an inability to tolerate medical therapy. Therefore, the risk of hemodynamic deterioration with expectant management was assessed as being very high and a safe emergent delivery would have been nearly impossible due to the combined cardiac and obstetric complications. The second option was urgent valve intervention while the pregnancy was maintained followed by expectant management. PMBV was not feasible due to the presence of significant valvular calcifications, so surgical valve replacement with cardio-pulmonary bypass would have been required. However, because of the diagnosis of PASD, the risk of life-threatening uterine hemorrhage with the doses of anticoagulation required for cardio-pulmonary bypass was assessed as prohibitively high. Therefore, delivery and hysterectomy were necessary before cardiac surgery. The third option considered and ultimately recommended to the patient was ECMO-supported caesarean section delivery followed by hysterectomy and subsequent valve replacement surgery.

For women of child-bearing age, selection of prosthetic valve type is nuanced.^11^ Mechanical valves require life-long anticoagulation and are associated with adverse fetal outcomes as well as maternal bleeding and thromboembolic complications. Bioprosthetic prostheses are more likely to result in a successful pregnancy outcome but are less durable, particularly in young adults in whom rates of structural valve degeneration are accelerated. In general, bioprosthetic valves are recommended over mechanical valves to optimize maternal and fetal outcomes during pregnancy (COR: IIa, LOE: C).^3^ In this case, the surgical team presented both options to the patient and explained the risks and benefits associated with each. Ultimately, she opted for bioprosthetic valves due to a strong desire to avoid life-long anticoagulation management as well as the higher short-term risk of bleeding complications with the anticoagulation required for mechanical valves having just undergone major open abdominal and cardiac surgeries.

We hope the clinical considerations in this case will inform clinicians in the management of severe MS during pregnancy and more generally reinforce the ESC guideline recommendations for pre-pregnancy counseling with a multidisciplinary pregnancy heart team for all women at risk of cardiac complications.

## Supplementary Material

ytae055_Supplementary_Data

## Data Availability

All available data are presented within the manuscript.

## Lead author biography

Dr. Jaya Batra is a Fellow in Cardiovascular Medicine at Columbia University Irving Medical Center at New York Presbyterian in New York City. Her clinical interests include valvular heart disease and advanced heart failure.
